# N-glycan PK Profiling Using a High Sensitivity nanoLCMS Work-Flow with Heavy Stable Isotope Labeled Internal Standard and Application to a Preclinical Study of an IgG1 Biopharmaceutical

**DOI:** 10.1007/s11095-015-1724-0

**Published:** 2015-05-28

**Authors:** Fabian Higel, Andreas Seidl, Uwe Demelbauer, Margot Viertlboeck-Schudy, Vera Koppenburg, Ulrich Kronthaler, Fritz Sörgel, Wolfgang Frieß

**Affiliations:** Analytical Characterization, Sandoz Biopharmaceuticals, HEXAL AG, Keltenring 1+3, 82041 Oberhaching, Germany; Clinical Bioanalytics, Sandoz Biopharmaceuticals, HEXAL AG, Oberhaching, Germany; Clinical R&D, Sandoz Biopharmaceuticals, HEXAL AG, Holzkirchen, Germany; IBMP - Institute for Biomedical and Pharmaceutical Research, Nürnberg-Heroldsberg, Germany; Institute of Pharmacology, Faculty of Medicine, University Duisburg-Essen, Essen, Germany; Department of Pharmacy, Pharmaceutical Technology and Biopharmaceutics, Ludwig Maximilians-Universität München, Munich, Germany; Process Analytics, Sandoz Biopharmaceuticals, Schaftenau, Austria

**Keywords:** high mannose, mass spectrometry, monoclonal antibody, N-glycosylation, pharmacokinetics

## Abstract

**Purpose:**

In this study an innovative, highly sensitive work-flow is presented that allows the analysis of a possible influence of individual glyco-variants on pharmacokinetics already during pre-clinical development. Possible effects on the pharmacokinetics caused by glyco-variants have been subject of several studies with in part contradictory results which can be related to differences in the set-up.

**Methods:**

Using 96-well plate based affinity purification an IgG1 antibody was isolated from preclinical samples and glycans were analyzed individually by nanoLCMS. Prerequisite was a reference standard based on stable heavy isotope labeled glycans. The high sensitivity and low sample consumption enabled the integration into the preclinical development program.

**Results:**

The data of an IgG1 biopharmaceutical from a preclinical rabbit study showed that some N-glycoforms have a different PK profile compared with the average of all molecule variants as determined by ELISA. IgG1 high mannose glycoforms M5 and M6 were removed from circulation at a higher rate.

**Conclusion:**

The results of the preclinical study demonstrated the applicability of the developed innovative workflow. The PK profile of glyco-variants could be determined individually. It was concluded that M6 was converted by mannosidases in circulation to M5 which in turn was selectively cleared by mannose receptor binding which is in-line with previously published results. Therefore the developed technology delivers reliable results and can be applied for PK profiling of other mAbs and other types of biopharmaceuticals.

**Electronic supplementary material:**

The online version of this article (doi:10.1007/s11095-015-1724-0) contains supplementary material, which is available to authorized users.

## INTRODUCTION

N-glycosylation, one of the most complex post-translational modifications is under suspicion to influence the pharmacokinetics of therapeutic proteins. One class of therapeutic proteins, mAbs that carry one N-glycan per heavy chain at their Fc part were subject of several studies.(1–3) For example Newkirk *et al*. generated degalactosylated IgG1 and IgG2 and showed that the degalactosylated mAb was cleared at a slower rate in mice.(4) In another study an IgG enriched in high mannose glyco-structures was compared to complex glycosylated IgG. No difference in PK was found by ELISA. Fab glycosylation was investigated too via enrichment of an IgG to contain one N-glycosylation in its variable domain. However no influence on PK was observed.(5) The major drawback of enrichment experiments is the generation of an artificial new IgG N-glycoform pattern that does not necessarily give the same results as the individual glycoforms in the original IgG. LC-MS based approaches allow the direct analysis of the N-glycosylation from the heterogenic mAb N-glycoform mixture. Using an LC-MS method an increasing clearance of high mannose N-glycans was reported by several groups.(6–10) With a single subject case study *Chen et al.* were the first to describe a conversion of high mannose glycans M6-M9 to M5 on an IgG2 which occurred in the blood stream due to circulating glycosidases.(6) The same group showed for an IgG1 an increased clearance of M5 glycoforms in addition to this conversion.(7) *Alessandri et al.* reported a conversion of M6 and M7 to M5 in circulation *in vitro* and increased *in vivo* clearance of the high mannose glycans compared to complex structures.(9) Using mAbs exclusively glycosylated with M8/9 or M5, studies were performed in mice showing that M8/9 and M5 IgGs were cleared much faster compared to complex glycosylated IgGs and that M8/9 glycans were *in vivo* converted to M6.(8) It was concluded that these faster cleared N-glycans bound to N-glycan specific receptors and were subsequently removed via endocytosis.(11–13) Correspondingly, the mannose specific receptor was thought to bind terminal mannose residues resulting in fast clearance of high mannose structures.(14–18) In contrast to the immunoassay and studies with enriched IgG fractions the results obtained with LC-MS methods from the human case studies point in the same direction. However, the published studies have some drawbacks such as the small number of subjects which does not allow statistically significant conclusions or the serum sample volumes do prevent the use of the study setup N-glycan PK profiling as an accompanying analysis to preclinical studies. The sample volumes during clinical studies could be sufficient to generate also data on N-glycoforms, but clinical studies are performed late from a development perspective and an adjustment of the mAb N-glycosylation based on the findings from the N-glycan PK profiling is hardly possible at this development stage. Therefore, it would be advantageous if the influence of N-glycans could be studied earlier, e.g., already during the preclinical phase or even in exploratory preclinical studies to be able to adjust critical quality attributes, for example by glyco-engineering. The prerequisites for this approach are that the observed effects during preclinical studies like the increased clearance of high mannose glycans must be representative for the results obtained in clinical studies Furthermore, serum sample consumption must be minimal and the sensitivity of the LC-MS methods must be increased drastically as serum volume is more limited in animal studies compared to human studies. Finally, the throughput of the methods must be increased to analyze a statistically powerful number of animals. In this study an analytical approach circumventing the mentioned limitations is presented. The method comprises a high-throughput sample preparation procedure encompassing affinity purification using immobilized antigen and glycan processing. The work-flow was completely based on 96-well plate format. Analysis of 2-AA labeled N-glycans was performed with a highly sensitive nanoLCMS using reversed phase chromatography which was previously shown to be robust and sensitive.(19) Quantification by MS was achieved using a stable heavy isotope 2-AA label that contains six ^13^C atoms.(20) The use of this internal standard compensated for variations in the sample preparation and resulted in a higher precision of the nanoLCMS analysis. Together with the small scale sample preparation N-glycan PK data were obtained from 50 μl serum samples from an IgG1 s.c. PK study performed in rabbits.

## MATERIALS AND METHODS

### Materials

2-Aminobenzoic acid, ethanolamine, formic acid, picoline borane, DMSO, ^13^C aminobenzoic acid were from Sigma (Munich, Germany). PNGaseF was from Roche (Penzberg, Germany). Acetic acid, acetonitrile and hydrochloric acid were from Merck (Darmstadt, Germany). NHS activated sepharose, Sephadex® G-10 96-well plates and 96-well deep well plates were from GE Healthcare (Munich, Germany). Antigen was from Peprotech (Hamburg, Germany). Phosphate buffered saline was from Gibco/Life technologies (Darmstadt, Germany).

Multicreen THS HV filter plates were from Milipore. IdeS protease was Genovis (Lund, Sweden). 96-well plates were from Nunc/Thermo Scientific (Munich, Germany). AcroPrep™ Advance Omega™ 10K 96-well filter plates were from Pall (Dreieich, Germany). Preclinical rabbit serum samples were obtained from clinical bioanalytics at Sandoz.

### Methods

#### Preclinical Rabbit Study

The preclinical study was performed in New Zealand White (NZW) rabbits. Following single subcutaneous administration of 10 mg kg^−1^ b.w. of an IgG1 biopharmaceutical (mAb1) blood samples were drawn from the *vena cephalica* or *saphena magna* over 29 days including one pre-dose blood sample. Detailed sampling is listed in Table I. Samples were stored at −70°C and were thawed at romm temperature immediately before analysis. Concentration of mAb1 in preclinical serum samples was determined using a sandwich ELISA. Free mAb1 was quantitatively analyzed using immobilized antigen and a horse radish peroxidase conjugated probe specifically binding mAb1 for detection. From remaining serum with one freeze thaw cycle 2 × 50 μl aliquots were used for glycan PK profiling. The first aliquot was analyzed and the second aliquot served as back-up aliquot and was stored at −70°C.

**Table I:** Sampling Schedule of the Preclinical Study of an IgG1. At Each Sampling Time Point ~500 μl of Serum Were Drawn

Day	1	1	1	2	2	3	3	4	5	8	15	22	29
Hours [post-dose]	0 (pre-dose)	2	8	24	40	48	60	72	96	168	336	504	672

#### Preparation of ^13^C 2-AA Labeled Glycan Standard

N-glycans of desalted mAb were released, labeled with [^13^C] 2-AA which contains 6 ^13^C atoms and purified as described earlier.(19) Purified ^13^C 2-AA labeled N-glycans (25 pmol/μl) were aliquoted and stored at −20°C until use.

#### Preparation of 96-Well Plate Affinity Columns with Immobilized Antigen

Antigen was dissolved in H_2_O (1 mg/mL), reconstituted for 2 h at room temperature and further. diluted (0.5 mg/ml) with H_2_O, aliquoted and stored at −20°C until use. Prior to immobilization antigen solution was prepared by diluting reconstituted antigen with PBS to a final concentration of 0.05 mg/ml. The membranes of a 96 well filter plate were wetted with 1 mM HCl (100 μL) before addition of 200 μL NHS activated sepharose-isopropanol slurry per well. Isopropanol was removed by centrifugation (100 g) and the columns were washed with 1 mM HCl (150 μL) for four times. Antigen solution (100 μL) was centrifuged (50 g) into the prepared columns and coupling reaction was allowed to take place for 2 h at ambient temperature. Affinity columns were washed by centrifugation (50 g) and remaining NHS groups were inactivated using ethanolamine buffer (150 μL). Finally microplate columns were equilibrated by centrifugation (50 g) with PBS.

#### Affinity Purification of an IgG1 Biopharmaceutical and Glycan Processing

Serum samples (50 μL) were diluted with PBS (50 μl) and applied to the affinity purification column by centrifugation (50 g). Antigen bound mAb1 was washed several times with PBS to remove serum and unspecific bound proteins. IdeS protease solution was centrifuged (50 g) into the columns to release the glycosylated Fc part of the mAb. Reaction was performed at 37°C for 30 min. Released Fc parts were eluted with PBS (2 × 150 μl) by centrifugation (100 g). Flow through was collected in new microplates. PNGaseF and ^13^C-2-AA labeled N-glycan standards were added. This mixture was incubated for 17 h at 37°C to release mAb1 N-glycans. N-glycans were purified by ultrafiltration using 96-well plates with 10K cut-off membranes. N-glycans (released N-glycans and ^13^C 2-AA glycan standard) were dried by vacuum centrifugation.

Dried N-glycans were dissolved in H_2_O (10 μL) and ^12^C 2-AA labeling solution (15 μL; 100 mg/mL picoline borane, 50 mg/mL 2-AA in a 7:3 mixture of DMSO and acetic acid) was added to label released mAb1 sample N-glycans. Labeling was performed at 37°C for 17 h.

Excess label was removed by gelfiltration using custom made 96-well Sephadex G-10 plates. Columns were equilibrated with 4 × 200 μL H_2_O by centrifugation (100 g). Labeled samples were filled up to 100 μL with H_2_O and applied to the gel filtration columns. 2-AA and ^13^C 2-AA labeled N-glycans were eluted with 150 μl H_2_O. Purified glycans were brought to dryness by vacuum centrifugation and were redissolved in 20 μL H_2_O for nanoLC-MS analysis.

#### nanoLCMS of Labeled N-glycans

NanoLCMS was performed as described earlier.(21) The light to heavy ratio (L/H) of the co-ionizing 2-AA N-glycans and the 6 Da heavier ^13^C 2-AA N-glycan standard was determined from the intensities of the monoisotopic peaks. The L/H was determined for each mAb1 N-glycan and its corresponding standard. By plotting the L/H ratio over sampling time points the glycan PK profile was obtained for the individual N-glycans. After normalization to the maximal L/H ratio the glycan PK profiles can be compared to the normalized ELISA profile. PK parameters (AUC, t1/2, etc.) were determined and statistical analysis of the data was performed using Graphpad Prism V6.04.

## RESULTS

### Preclinical Study and ELISA

**Fig. 1:**
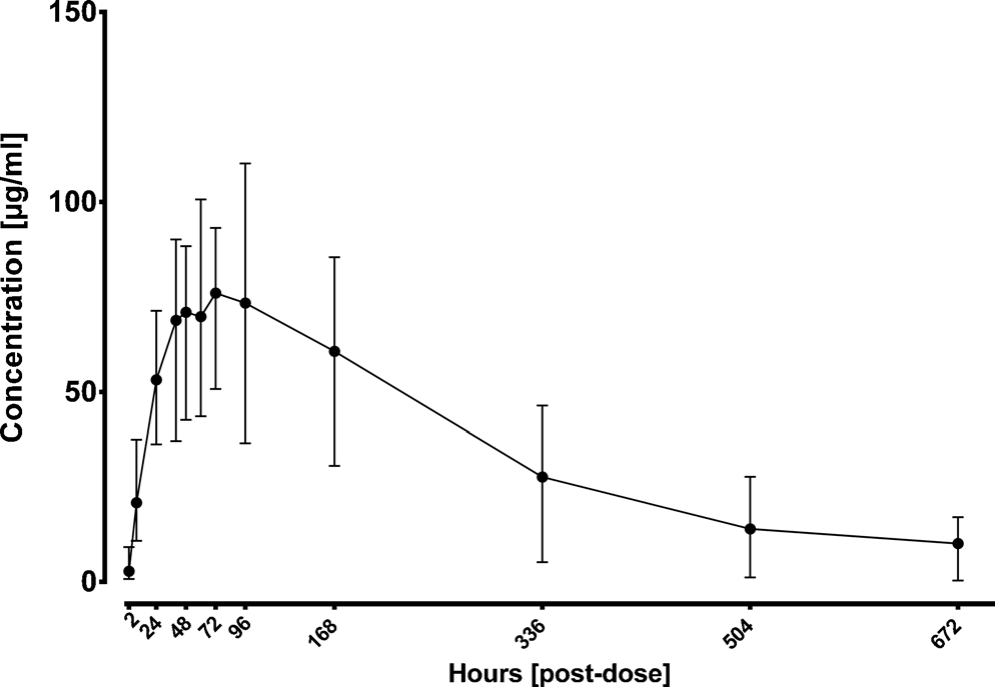
ELISA results with range from 15 rabbits that were included in the preclinical study of an IgG1 biopharmaceutical.

The preclinical study was performed in New Zealand White Rabbits. 15 animals were included in this study. A single subcutaneous dose of an IgG1 biopharmaceutical (mAb1) with 10 mg per kg body weight was administered. Serum samples were taken at 12 sampling time points after administration (Table I). Concentration of mAb1 was determined by ELISA. (Fig. 1). Remaining serum samples (50 μl) were used for N-glycan PK profiling.

### N-glycan PK Profiling Method Development and Qualification

**Fig. 2:**
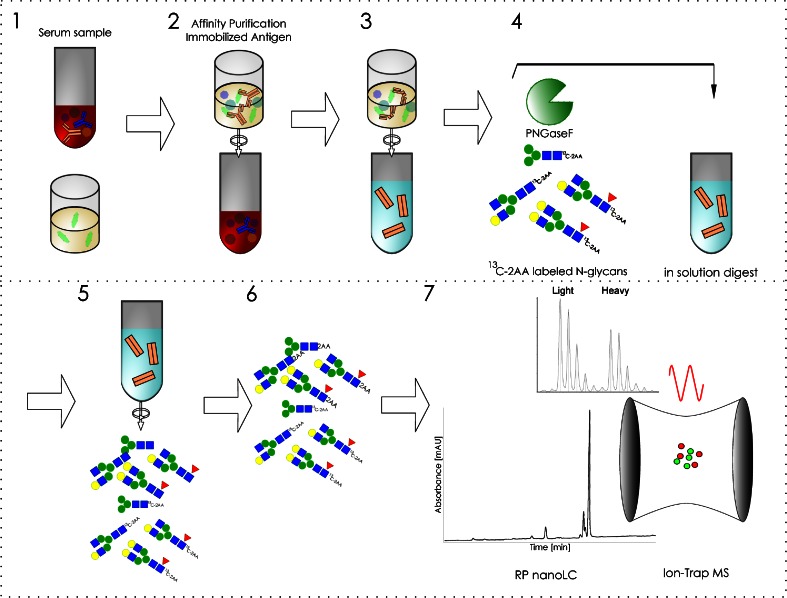
Glycan PK profiling work-flow. *1*. Serum samples are applied to 96-well based affinity purification columns with immobilized antigen (*light green*). *2*. The column is extensively washed. *3*. The N-glycan containing Fc part is released by centrifugation of IdeS enzyme into the column and incubation at 37°C for 30 min. Fab and unspecific bound proteins remain on the column. *4*. PNGaseF and heavy isotope standard (^13^C 2-AA N-glycans) are added to the Fc part. Incubation for 17 h at 37°C. *5*. Released sample N-glycans and heavy isotope standard are purified from proteins by ultrafiltration. *6*. 2-AA labeling of sample glycans and subsequent gelfiltration to remove excess label. *7*. NanoLC-MS analysis.

To be able to analyze the different N-glycans of mAb1 from serum samples the antibody had to be affinity purified and therefore a workflow had been developed which is described briefly in the following: Affinity columns consisting of NHS sepharose were prepared in 96 well filter plates. Then 5 μg mAb1 antigen per well were immobilized by covalent binding to the NHS groups. Remaining free NHS groups were deactivated with ethanolamine buffer and equilibrated with PBS. Recovery of mAb1 from rabbit serum is schematically shown in Fig. 2. Preclinical serum samples were applied to the affinity columns. The samples were centrifuged through the column and the resin was washed several times with PBS to remove unspecific bound serum proteins. To obtain pure glycan samples the glycan containing Fc part of the immobilized antibody was subsequently specifically released using IdeS enzyme. The eluted antibody fragments were deglycosylatied by use of PNGaseF. Together with PNGaseF a stable heavy isotope standard, ^13^C 2-AA labeled N-glycans obtained from mAb1 was added to the samples. Released sample N-glycans were then labeled with 2-AA and excess label was subsequently removed by gelfiltration. Labeled N-glycans were analyzed by nanoLC-MS and the ratio of the peak areas of light sample glycan and its corresponding heavy isotope standard were determined. This light to heavy ratio (L/H) was determined for each N-glycan for each sampling time point and animal. By plotting the average L/H ratio from all 15 animals against time individual PK profiles for each N-glycan were obtained. Glycan maps were calculated for each sampling time point based on the known L/H ratios and the relative distribution of the heavy isotope standard glycans which was determined in a separate nanoLCMS run. To qualify the method for its intended purpose several criteria were determined. The qualification of the nanoLCMS method was described previously (21). With the determined maximal antigen binding capacity of 5 μg in the 96-well plates at theoretical ULOQ of 15 μg mAb1 or 250 μg/ml using 50 μl serum could be calculated. Next LOD, LLOQ and linearity were determined for each N-glycan in dilution series of mAb1 in neat serum (Table S1). The LLOQ (10 μg/ml) was approximately three times higher than the LOD (3 μg/ml). Linearity was determined for each N-glycan individually with maximal concentrations between 100 and 200 μg/ml (Table S1). Linearity (R^2^ > 0.98) plots are depicted in supplementary Figure S1. Robustness of the method was assessed by comparing the results of two operators (Figure S2). Matrix interference was excluded by analyzing mAb1 N-glycans after spiking into and purification from different animal (rabbit, monkey) and human sera. Accuracy was determined by comparing results of the glycan maps of mAb1 after affinity purification to the one obtained directly from the drug product (Figure S3). In order to test the method for the detection of changes of single N-glycans a proof of concept study was performed by spiking mixtures of mAb1 with increasing portions of degalactosylated mAb1 into rabbit serum and using the developed method to detect the differences (Table S2 and Figure S4). The first experiment contained exclusively untreated mAb1 and served as control experiment. Experiment 1 had therefore constant galactosylation of 18%. In the second experiment increasing portions of degalactosylated mAb1 were mixed with untreated mAb1 starting from 0 to 100% degalactosylated mAb1. Small levels of terminal galactosylation were detected in the sample containing 100% degalatosylated mAb1 indicating incomplete degalactosylation. The third dilution series reflected decreasing galactosylation from 18% to approximately 15% which corresponds a relative decrease by 15%. Considering an incomplete degalactosylation as seen for experiment 2 this result demonstrated that a relative decrease of 15% terminal galactosylation can be detected. In experiment 4 where a decrease of 5% galactosylation was prepared no significant decrease compared to the control was observed. Thus the decrease of individual N-glycans should be at least 10–15% (relative to initial portion) in order to be detected with the developed approach. The linear range between 10 and 100 μg/ml when using 50 μl of serum samples was sufficient to analyze the rabbit study.

### mAb1 N-glycosylation and Qualification of the Study

**Fig. 3:**
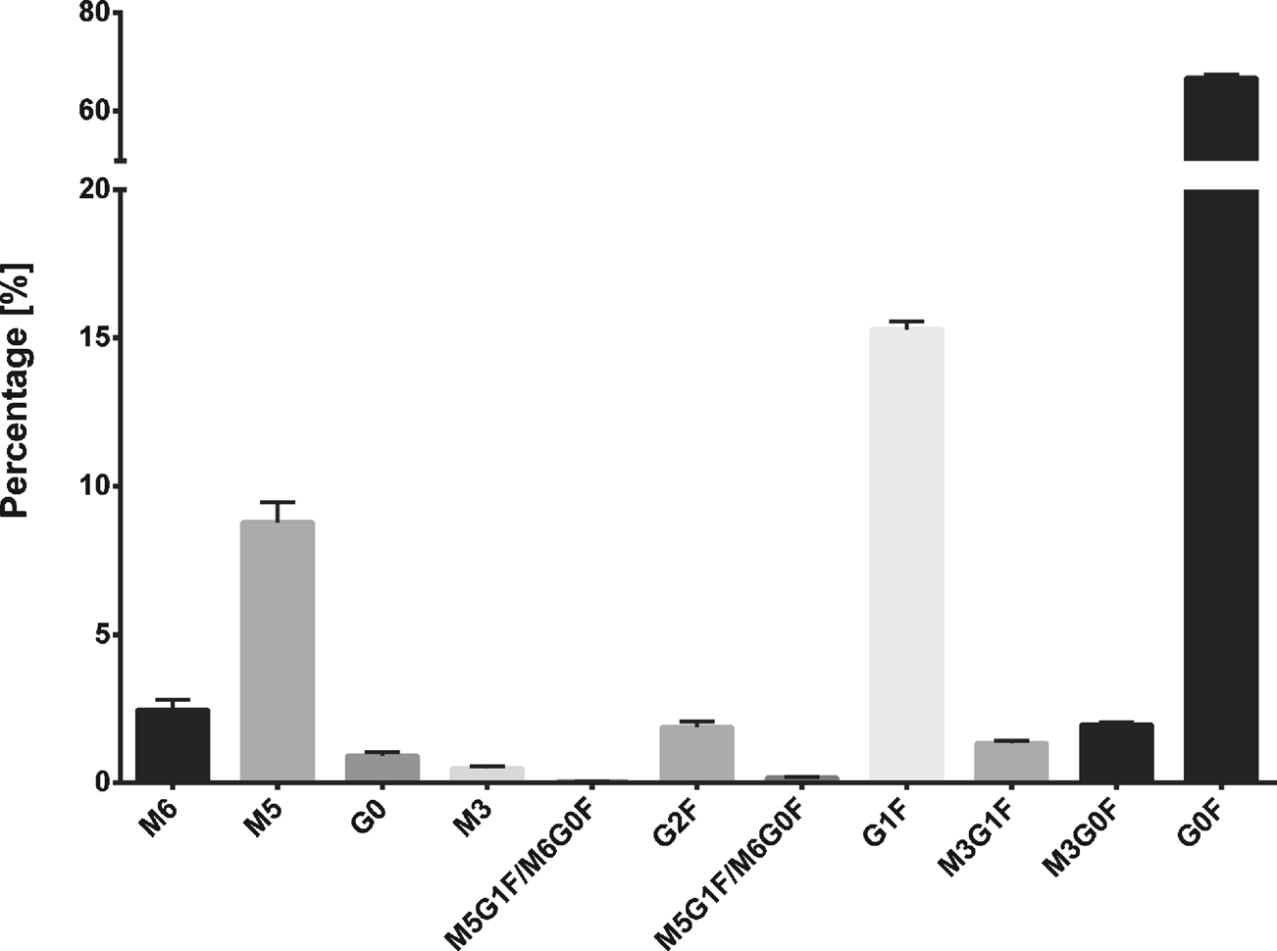
Average glycan map obtained from 10; 50 and 100 μg/ml quality check samples shows the relative N-glycan composition. Error bars show the standard error.

**Fig. 4:**
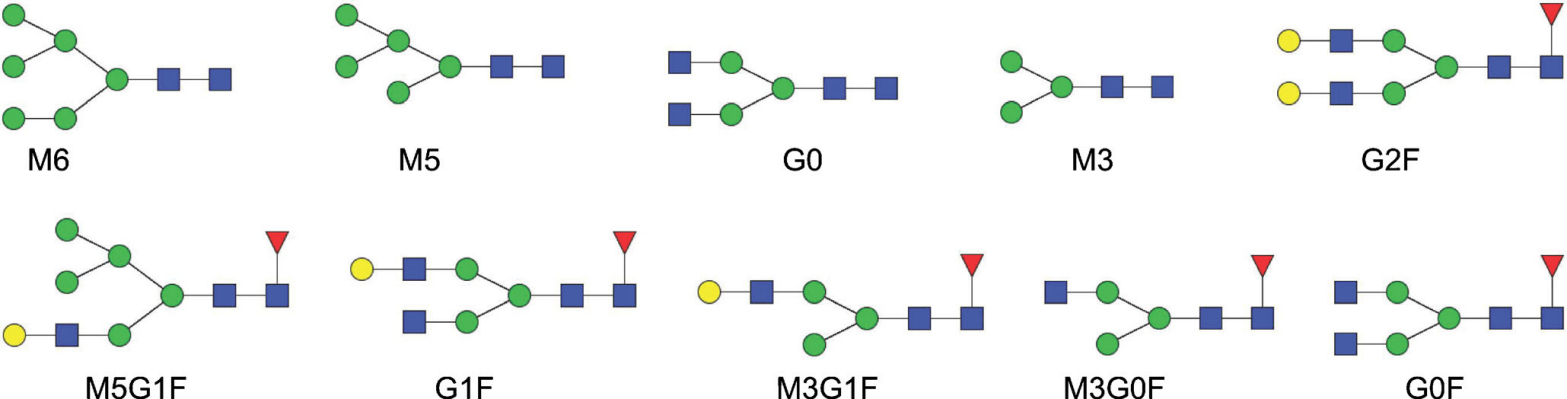
N-glycan structures of mAb1. *Blue square* stands for N-Acetylglucosamine, *green circle* for mannose, *yellow circle* for galactose and *red triangle* for fucose.

To control the affinity purification quality check samples were prepared by spiking known amounts of mAb1 into NZW rabbit serum. The quality check samples covered the concentration range of the study which was previously determined by ELISA. Figure 3 shows the mean glycan maps of the 10, 50 and 100 μg/ml mAb1 quality check samples. With the developed enzymatic elution of the glycosylated antibody fragments very high selectivity and purity was achieved. No interfering N-glycans of mAb1 were co-purified with the exception of two minor abundant bisecting variants that were excluded from analysis. No additional serum related N-glycans were detected. Sensitivity was sufficient to analyze all N-glycans with a percentage of at least 0.1% at a concentration of 10 μg/ml. The N-glycans were mainly complex biantennary with core fucose. The most abundant N-glycan was G0F (65%) with terminal N-acetylglucosamine residues followed by G1F (16%) with one additional terminal galactose. High mannose glycan M5 (9.5%) was the third most abundant N-glycan. All other N-glycans had a portion of less than 3%. mAb1 contained no N-glycans with terminal sialic acids. Glycan structures are shown in Fig. 4.

### Glycan PK Profiles of mAb1

**Fig. 5:**
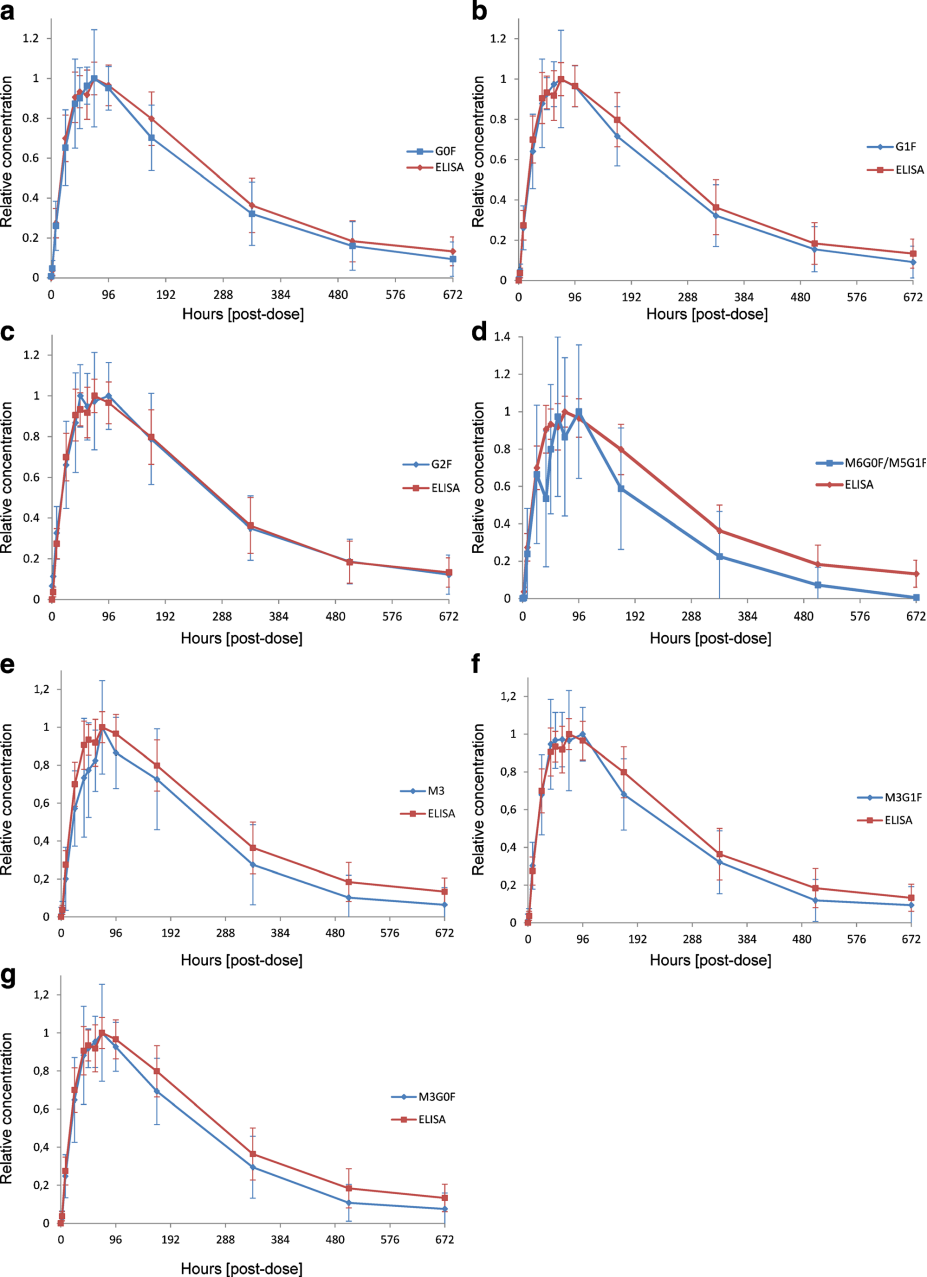
Comparison of nanoLC-MS based glycan PK data (*blue*) and ELISA data (*red*) for G0F (**a**), G1F (**b**), G2F (**c**), M5G1F/M6G0F (**d**), M3 (**e**), M3G1F (**f**), M3G0F (**g**). Concentration is relative to the maximum of each curve to enable comparison. Mean profiles from 15 animals are shown.

**Fig. 6:**
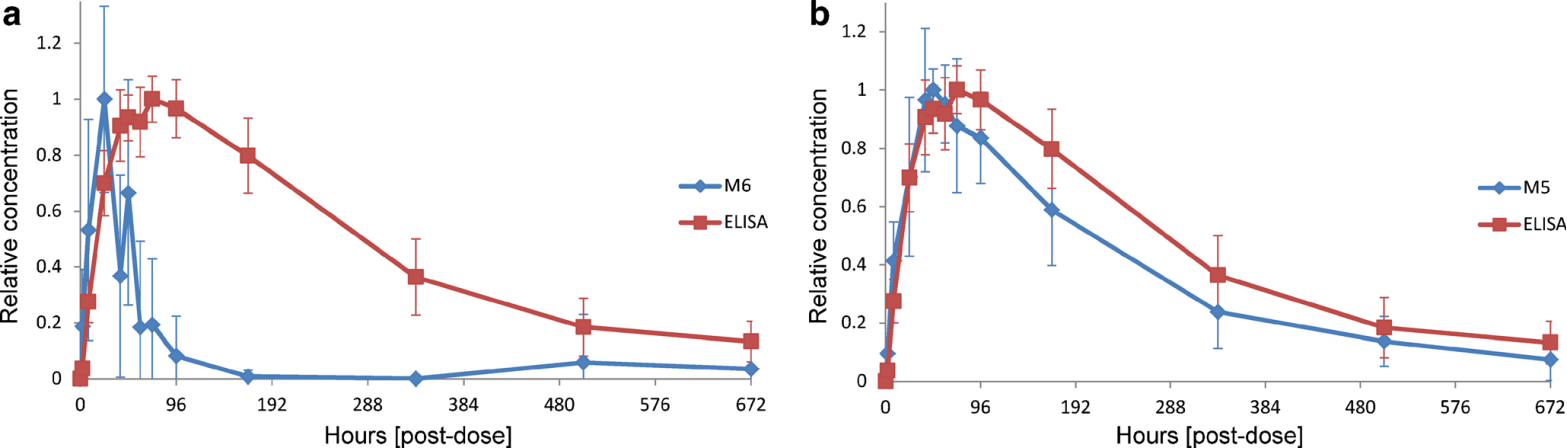
Comparison of high mannose glycan M6 (**a**) and M5 (**b**) PK profiles obtained by nanoLC-MS (*diamond*) and ELISA (*squares*) profiles.

The determined glycan L/H ratios were plotted against the sampling time to obtain individual PK profiles for each N-glycan. Mean L/H ratios for each N-glycan were normalized to the maximum of each N-glycan L/H curve to obtain a relative concentration (Fig. 5). ELISA data were normalized in the same way. The PK profile of the most abundant complex type G0F was very similar to the ELISA profile (Fig. 5a). The maximum concentration (t_max_) was reached after 72 h with congruency of the ELISA and the L/H glycan graph. Elimination occured at identical rates and the PK profiles were regarded as comparable. This finding was expected as the ELISA represents the average profile of all mAb1 glycoforms and G0F being the major N-glycan representing the majority of these glycoforms. The second most abundant complex type N-glycan G1F that accounted for approximately 16% of all mAb1 N-glycans also showed a PK profile which was very similar compared to the ELISA profile (Fig. 5b). Again t_max_ was reached at 72 h. Complex type G2F with a portion of 2% had the best match with the ELISA profile showing almost perfect congruency (Fig. 5c). The profile of the M6G0F/M5G1F hybrid type glycan represented two isomers that could not be differentiated with the nanoLC-MS approach and which had a relative content of only 0.1%. The profile and t_max_ were similar to the ELISA profile as well (Fig. 5d). The low abundance brought the analysis close to the LLOQ which resulted in lower precision and higher variation. The glycan PK profiles of M3, M3G0F and M3G1F were also highly similar to the ELISA profile (Fig. 5e, f and g). These results demonstrated that PK profiles could be obtained for each N-glycan individually. The complex glycan PK profiles were highly similar to the ELISA for the most abundant glycans. For N-glycans with a portion smaller than 0.5% the graphs showed higher variation. Considering all the glycan forms analyzed, only the high mannose type N-glycans M5 and M6 with 9.5 and 2% relative abundance respectively showed a clear discrepancy from the ELISA profile (Fig. 6) The maximum concentration of M6 was reached after 24 h followed by either a conversion to M5 or an increased elimination rate that led to a faster clearance to a level below the LLOD or a complete removal from circulation at the 168 h time point. The high mannose glycan M5 profile also differed from the overall mAb ELISA PK profile (Fig. 6b). The t_max_ was reached 24 h earlier already after 48 h and these molecules were cleared faster between 48 and 168 h. PK profiles for G0 could not be obtained due to co-elution of a contaminant with the same m/z value. The tmax values are listed in Table II.

**Table II:** Pharmacokinetic Parameters of Individual N-glycans Obtained from PK Profiles. The Time Until the Maximal Concentration (*tmax*), the Elimination Half-life (t_1/2_) and the Area Under the Curve (*AUC*) Were Determined for Each N-glycan and the ELISA from the Average of All 15 Animals. Relative AUCs of Each N-glycan Were Tested for Significantly Difference to the ELISA AUC with Unpaired t-tests

	M6	M5	M3	G2F	G1F	M3G1F	M3G0F	G0F	ELISA
tmax [h]	24	48	72	48	72	96	72	72	72
t1/2 [d]	0.7	12.6	13.0	14.5	13.9	13.3	13.3	13.8	14.8
Relative AUC [0-672]	44	214	231	258	257	231	233	256	270
Significant different to ELISA (*p* < 0.05)	Yes *p* < 0.0001	Yes *p* = 0.0175	No	No	No	No	No	No	ND

### Relative Decrease of M5 and M6

**Fig. 7:**
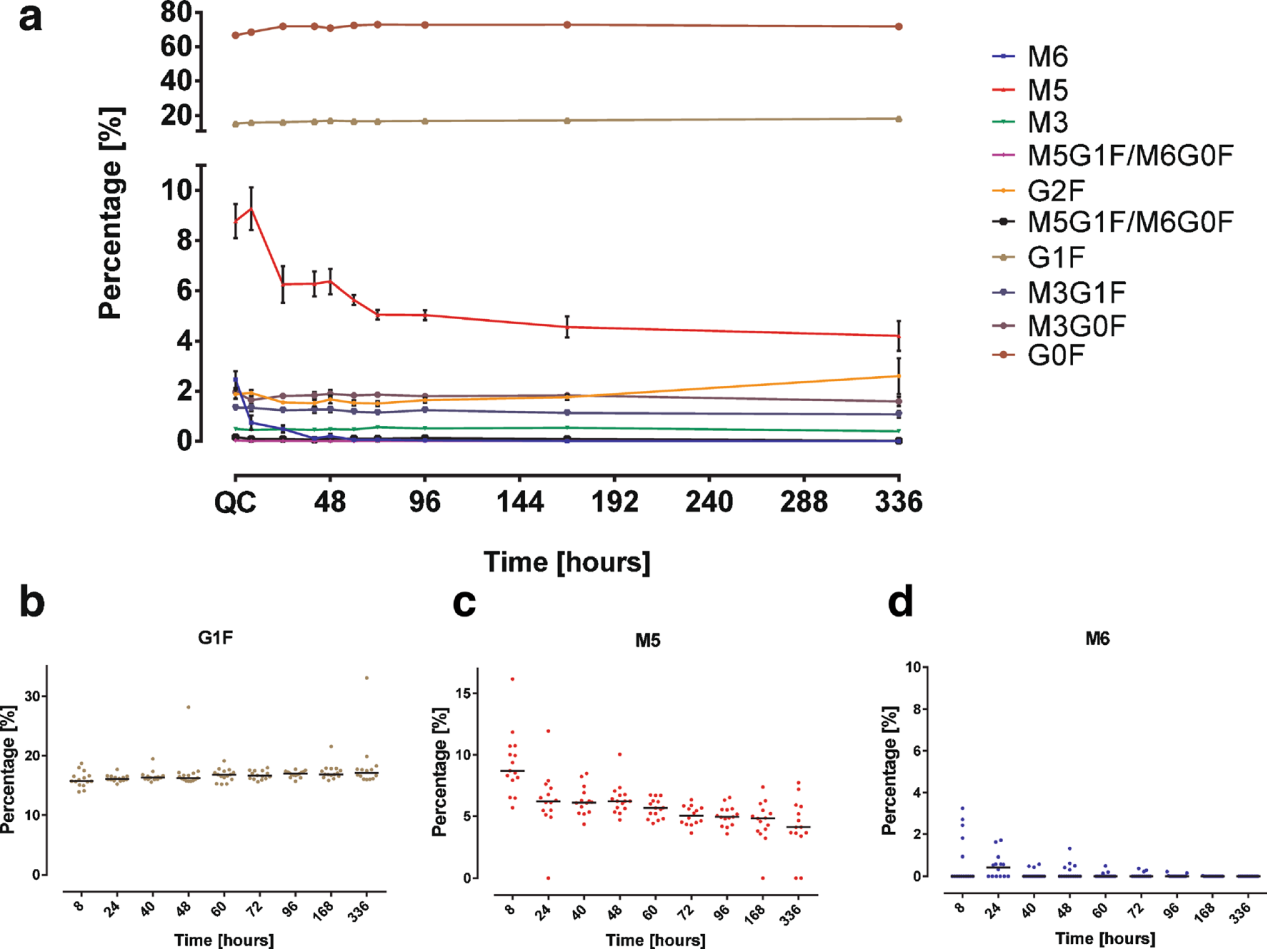
Glycan Maps of mAb1 for each time point. (**a**) Mean percentage and standard error of recovered mAb1 N-glycans after single subcutaneous administration in rabbits. The glycan map of the spiked QC samples is shown as reference. *Dot plots* showing the glycan percentages of individual rabbits for G1F (**b**), M5 (**c**) and M6 (**d**).

As shown above high mannose glycan PK profiles differed from the ELISA profile of mAb1 which represented the average concentration of all protein variants. These findings are reflected in the glycan maps calculated for sampling time points between 8 and 336 h (Fig. 7a).. At 2, 504 and 672 h the mAb1 serum concentration was below the LLOQ of 10 μg/ml. The glycan map of the QC samples as shown in Fig. 3 is depicted as well. Changes in the glycan composition of mAb 1 during circulation through selective clearance or conversion would result in a decrease or increase in the glycan map. Mean percentages of the most abundant N-glycans G0F and G1F stayed constant indicating no change over time which confirmed the previously made observations based on the glycan PK profiles (Fig. 7a). The glycan maps illustrate that the contribution of all N-glycans was constant except for the high mannose glycans M5 and M6 portions which decreased over time. The increase of G2F at 336 h was due to a single outlier in one animal. M6 was removed from circulation completely or below the LLOQ within 168 h whereas the M5 portion decreased after a small initial increase from the initial percentage of 9% to approximately 4% after 336 h. The value of M5 increase corresponds to the M6 decrease within the same time frame. Dot plots showing the percentages of M5 and M6 (Fig. 7c and d) for the individual rabbits demonstrate that the trending was observed in all animals and not due to single outliers. G1F was plotted as a constant reference (Fig. 7b).

The relative quantification of individual N-glycans and thereof obtained glycan PK profiles allow the determination of several pharmacokinetic parameters like tmax, t1/2 and the AUC from the mean PK profiles of all 15 animals (Table II). Values for the two M5G1F/M6G0F isomers were not determined because of the low abundance (~0.1%). The t_max_, t_1/2_ and AUC of the ELISA representing the average glycosylated molecule are depicted too serving as a reference. The t_max_ of the ELISA (72 h) is identical with the two most abundant glycans G0F and G1F. Elimination half-lifes are similar as well demonstrating the accuracy and precision of the glycan PK approach. M6 has the lowest t_max_ with 24 h followed by M5 and G2F (48 h). M6 and M5 had the lowest elimination half-life and the AUC. The differences reflect the observed decrease seen in the glycan maps. M6 is rapidly removed whereas M5 is removed at a slower rate. Relative AUCs of the individual N-glycans were tested with unpaired *t*-test for significance of differences (*p* < 0.05) compared to the ELISA. M5 (*p* = 0.0175) and M6 (*p* < 0.0001) were statistically significant different to the ELISA (Table II and Figure S5).

## DISCUSSION

An innovative approach for the investigation of individual N-glycan pharmacokinetics was utilized to analyze a preclinical rabbit study of an IgG1 biopharmaceutical. Using 96 well plates based high throughput affinity purification with the immobilized antigen and a stable heavy isotope standard nanoLC-MS quantification glycan it was possible to obtain PK data from only 50 μl serum samples for mAb concentrations between 10 and 90 μg/ml. A rabbit study including 15 animals was successfully analyzed and demonstrated the feasibility of the developed approach as an accompanying method in preclinical development. PK profiles for individual N-glycans were obtained which was so far not described in literature. These glycan PK profiles were compared to ELISA data demonstrating that high mannose glycans M5 and M6 had a statistically different PK profile. Glycan maps showed that M6 was either removed below the LLOQ or was completely removed during the first 48 h and M5 levels decreased from 9.5% to approximately 4%. These differences of M5 and M6 decrease were reflected in the elimination half-life and t_max_. The elimination half-life of M6 is only 0.7 days indicating a fast mechanism of removal. M5 has a longer elimination half-life yet still shorter than half-life of all other N-glycans indicating a different second mechanism of removal from the circulation. High mannose species M6-M9 are most likely converted by glycosidases in circulation to the smaller high mannose glycans M5 in humans (6,9). In mice a similar observation was made as high mannose species M7-M9 were converted to M6 (8). In the present study an initial increase of M5 and decrease of M6 was observed indicating a fast *in vivo* conversion from M6 to M5. However, these conversion mechanisms are not applicable for the M5 glycoform because of the enzyme specificity. Removal of mannose residues from M6-M9 requires cleavage of α 1,2 glycosidic bonds whereas M5 terminal mannose residues are connected via α 1,3 and α 1,6 glycosidic bonds. This is in agreement with the observation of constant M3; M4 glycans were not detected in the IgG used in the present study. The fast decrease in M5 can be explained by a second mechanism. M5 and in conclusion M5 containing glycoforms are probably removed by a specific clearance mechanism from circulation involving the mannose receptor which binds amongst others specifically to mannose containing glycoproteins and removes them from circulation with similar functions in humans and rabbits that were described previously (11,12,14,16,17,22). The incomplete removal of M5 from circulation observed in this study can be explained by the structural conformation of the Fc part. Several investigations showed that IgGs exhibiting N-glycans with terminal galactosylation on both heavy chains change the conformation of the Fc part to a horseshoe conformation which makes the N-glycans accessible for receptors (23). The mannose receptor is then able to bind M5 and remove the IgG molecules from circulation. It was shown that the assembly of the glycosylated heavy chains during protein biosynthesis in the ER is not random (24). For an IgG2 biopharmaceutical it was demonstrated that the glycoform M5:M5 is favored (7). For mAb1 in the present study a similar observation was made (Table III). Assuming random pairing all theoretical glycoforms percentages can be calculated which is shown in Table III for most abundant M5 containing glycoforms. MS data from intact mAb1 showed that the M5:M5 glycoform content was four times higher than the theoretical value assuming random heavy chain assembly. Thus M5:M5 was strongly favored during protein biosynthesis. The mAb1 Fc part with this glycoform combination may exhibit a horseshoe conformation which in turn results in an increased clearance from circulation by binding to the Mannose receptor. The remaining 4% M5 observed in the preclinical study were probably mAb1 molecules containing M5 in combination with G0F and other glycoforms that are small enough for a closed Fc conformation. Other glycoforms with an open Fc part could be M5:G1F or M5:G2F. However analysis of these glycoforms was not possible for mAb1 because of the major glycoforms overlaying the M5:G1F and M5:G2F glycoforms in the intact mass spectrum. Overall, the study results are similar to data from human case studies (6,7), which demonstrates the value of glycan PK profiling with preclinical samples. However, the observed disappearance of M6 appears to be contradictory to literature. It was reported that after an initial decrease of M6 by about 50% the levels remain fairly constant over time (6,9). It could be speculated that in the present case M6 disappears due to a different M6 removal or conversion mechanism in rabbits.

**Table III:** Portions of Glycoforms Containing M5. Theoretical Values Were Calculated Based on the Assumption of Random Pairing. Observed Values Were Obtained from MS Analysis of intact mAb1

	M5:M5	M5:G1F	M5:M3G1F	M5:G0F	M5:M3G0F	G0F:G0F
Calculated relative	0.62%	1.12%	0.11%	5.29%	0.18%	45.43%
Calculated relative to the most abundant G0F:G0F	1.36%	2.46%	0.24%	11.64%	0.40%	100%
Observed relative to the most abundant G0F:G0F	5.13%	ND	ND	12.73%	ND	100%

In summary, an innovative approach for glycan PK profiling of N-glycans was developed. Due to its high sensitivity samples from preclinical studies with low available sample volumes could be analyzed. The miniaturized work-flow in 96-well plates allows the preparation of many samples in parallel. The preclinical study results confirm previous findings from individual human case studies and therefore are representative for clinical studies. Thus the effect of N-glycosylation can be predicted by preclinical studies and detected earlier in biopharmaceutical development which allows optimizing N-glycosylation via glyco-engineering before entering clinical phases.

## Electronic supplementary material

ESM 1 (DOCX 597 kb)

## Abbreviations

2-AA: 2-Aminobenzoic acid
AUC: Area under the curve
DMSO: Dimethyl sulfoxide
EIC: Extracted ion chromatogram
ELISA: Enzyme linked immunosorbent assay
Fab: Fragment antigen binding
Fc: Fragment crystalizable
IgG: Immunoglobulin G
LC-MS: Liquid chromatography- Mass spectrometry
LLOQ: Lower limit of quantification
LOD: Limit of detection
NHS: N-hydroxysuccinimide
PBS: Phosphate buffered saline
PK: Pharmacokinetics
PNGaseF: Peptide N-Glycosidase F
t_1/2_: Elimination half-life
t_max_: Time until the maximal concentration is reached
ULOQ: Upper limit of quantification
