# Supplementary material for: N-glycan PK Profiling Using a High Sensitivity nanoLCMS Work-Flow with Heavy Stable Isotope Labeled Internal Standard and Application to a Preclinical Study of an IgG1 Biopharmaceutical
Source: Pharm Res. 2015 May 28;32(11):3649–59. doi: 10.1007/s11095-015-1724-0 (PMC4596906; doi:10.1007/s11095-015-1724-0)
Supplement: Supplementary file 1 — (DOCX 597 kb) [file 11095_2015_1724_MOESM1_ESM.docx]

***N-glycan PK Profiling Using a High Sensitivity nanoLCMS Work-Flow with Heavy Stable Isotope Labeled Internal Standard and Application to a Preclinical Study of an IgG1 Biopharmaceutical***

***Supplementary material***

Authors: Fabian Higel^1^, Andreas Seidl^1^, Uwe Demelbauer^7^, Margot Viertlboeck-Schudy^2^, Vera Koppenburg^2^, Ulrich Kronthaler^3^, Fritz Sörgel^4,5^, Wolfgang Frieß^6^

^1^ Analytical Characterization, Sandoz Biopharmaceuticals, HEXAL AG, Oberhaching, Germany

^2^ Clinical Bioanalytics, Sandoz Biopharmaceuticals, HEXAL AG, Oberhaching, Germany

^3^ Clinical R&D, Sandoz Biopharmaceuticals, HEXAL AG, Holzkirchen, Germany

^4^ IBMP - Institute for Biomedical and Pharmaceutical Research, Nürnberg-Heroldsberg, Germany

^5^ Institute of Pharmacology, Faculty of Medicine, University Duisburg-Essen, Essen, Germany

^6^ Department of Pharmacy, Pharmaceutical Technology and Biopharmaceutics, Ludwig Maximilians-Universität Muenchen, Munich, Germany

^7^ Process Analytics, Sandoz Biopharmaceuticals, Schaftenau, Austria

Table S1 Linear range with LOD and ULOQ determined from mAb1 dilution series in neat serum. Linear correlation was determined for each N-glycan individually. LOD and ULOQ for mAb1 are given by the LOD and ULOQ of the nine relevant (R²>0.980) N-glycans.

|  | **Linear range [µg/mL]** | | **Linear correlation** |
| --- | --- | --- | --- |
| **N-glycan** | LOD | ULOQ | R² |
| M6 | 3.1 | 100.0 | 0.996 |
| M5 | 0.8 | 100.0 | 0.991 |
| G0 | 1.6 | 100.0 | 0.984 |
| M3 | 3.1 | 200.0 | 0.986 |
| M5G1F/M6G0F | 25.0 | 100.0 | 0.979 |
| G2F | 0.8 | 200.0 | 0.985 |
| M5G1F/M6G0F | 12.5 | 100.0 | 0.971 |
| G1F | 0.8 | 100.0 | 0.992 |
| M3G1F | 0.8 | 100.0 | 0.988 |
| M3G0F | 1.6 | 100.0 | 0.995 |
| G0F | 0.8 | 100.0 | 0.992 |
| **mAb1** | **3.1** | **100.0** |  |


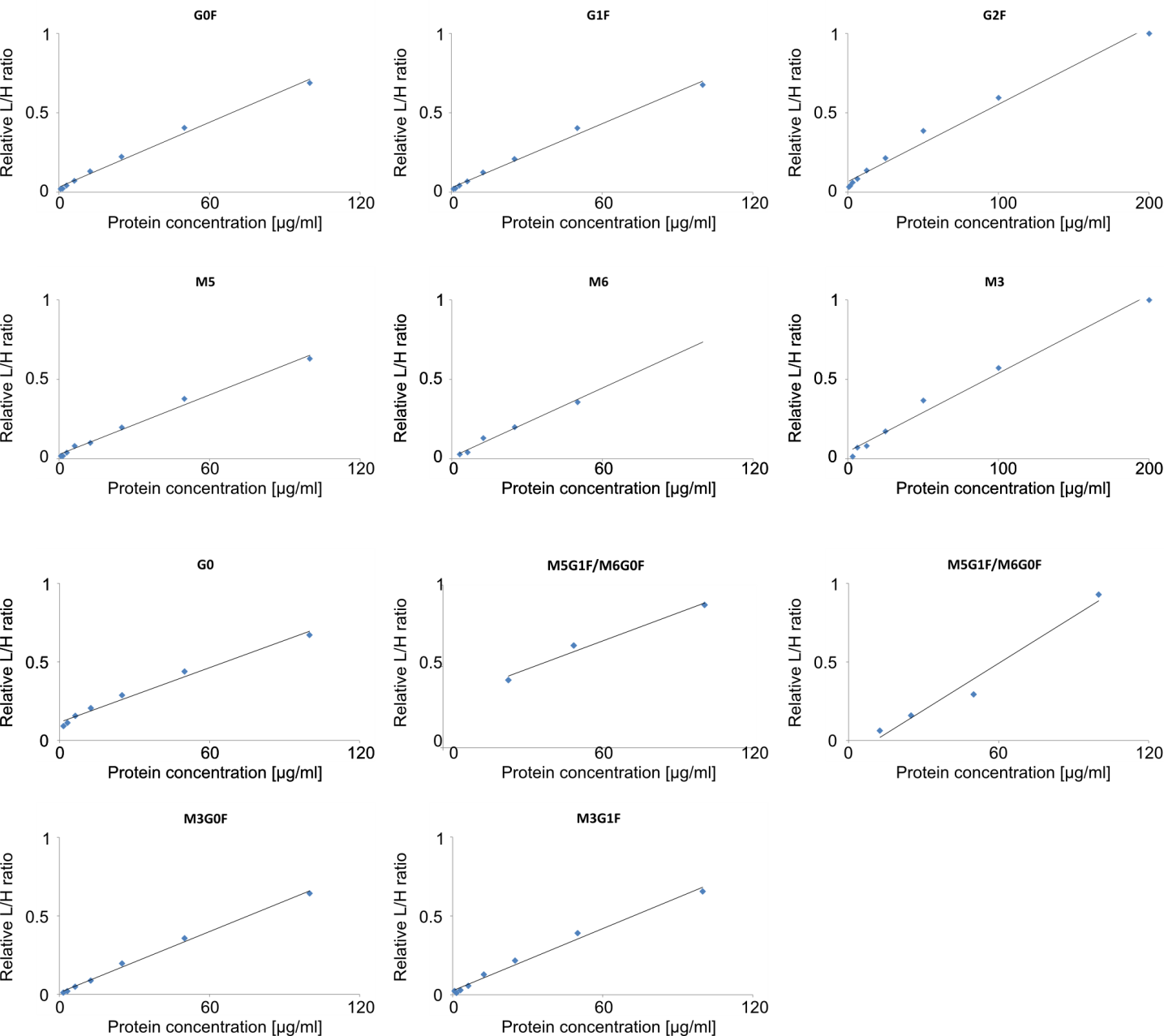


Figure S1 Linearity plots of mAb1 N-glycans. L/H ratios relative to the maximal concentration were determined for each mAb1 N-glycan individually with R²>0.98.


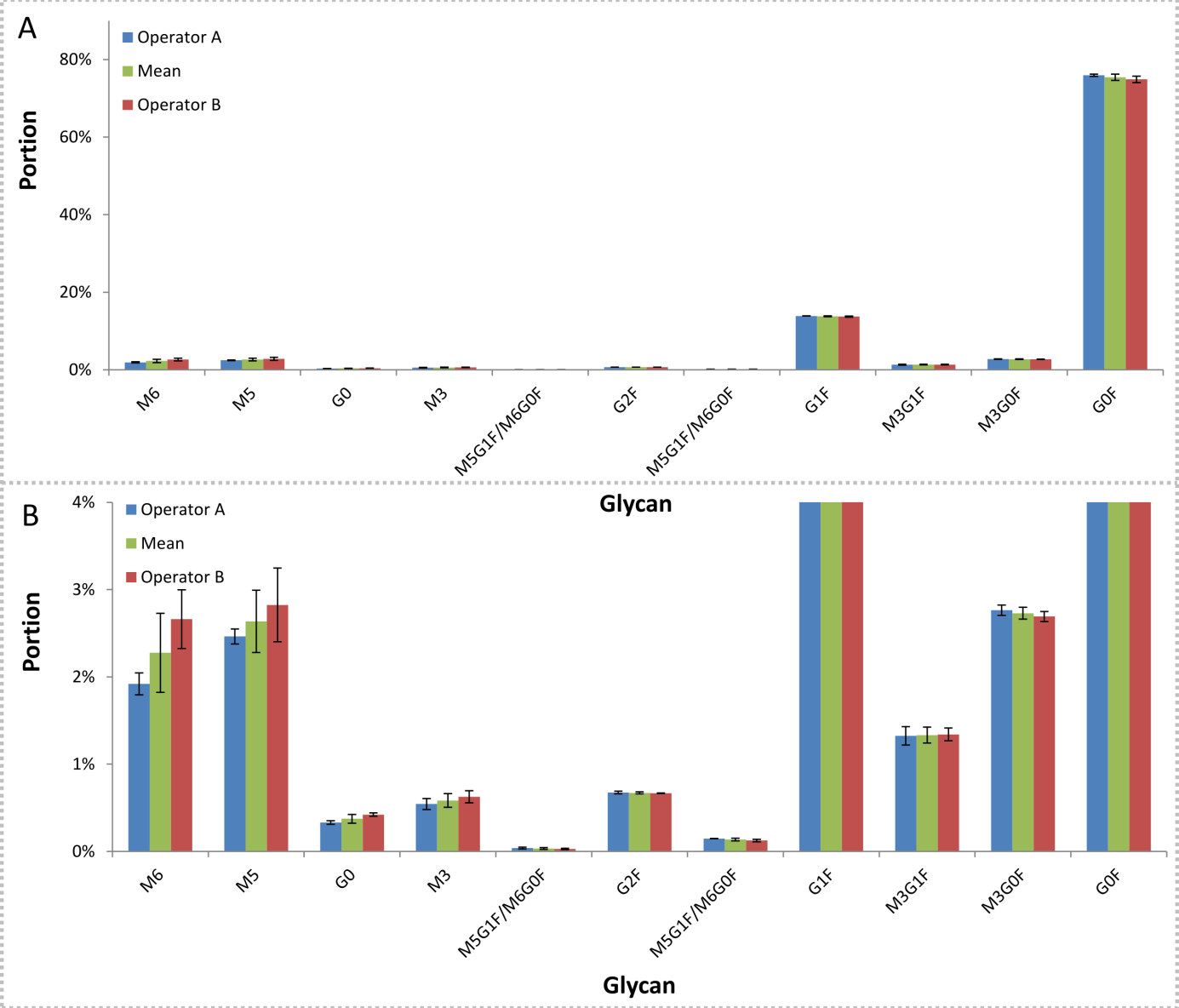


Figure S2 Robustness of the method. (A) Mean percentages with standard deviation of two operators who performed the glycan PK profiling method with spiked samples. (B) Magnified view shows minor abundant N-glycans.


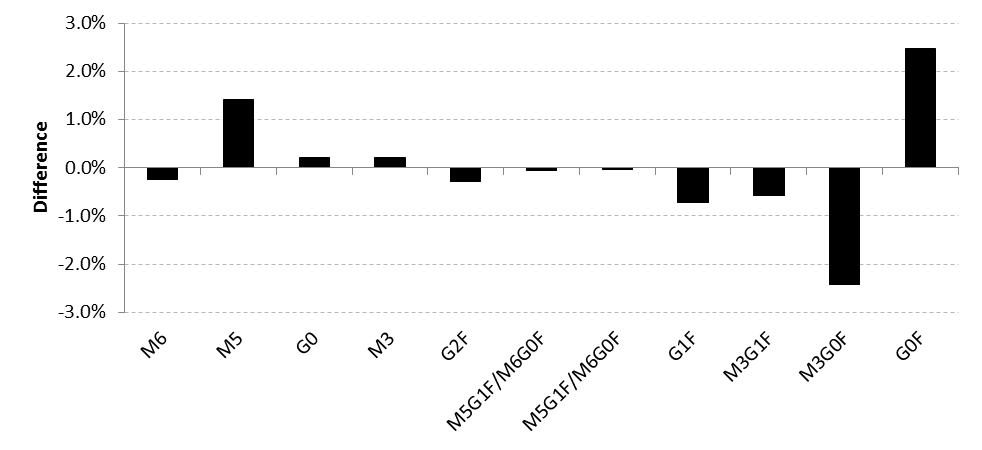


Figure S3 Difference plot for each glycan from glycan maps obtained from mAb1 after spiking and affinity purification from serum and mAb1 drug product.

Table S2 Proof of concept study. Degalactosylated mAb1 was spiked in four dilution series with untreated mAb1 to serum to test the developed work-flow. Total mAb1 concentration was constant (100 µg/ml).

|  | **Experiment 1** | | **Experiment 2** | | **Experiment 3** | | **Experiment 4** | |
| --- | --- | --- | --- | --- | --- | --- | --- | --- |
| **#** | **Deglactosylated mAb1** | **normal mAb1** | **Deglactosylated mAb1** | **normal mAb1** | **Deglactosylated mAb1** | **normal mAb1** | **Deglactosylated mAb1** | **normal mAb1** |
| **1** | 0,0% | 100,0% | 0,0% | 100,0% | 0,0% | 100,0% | 0,0% | 100,0% |
| **2** | 0,0% | 100,0% | 10,0% | 90,0% | 2,5% | 97,5% | 0,6% | 99,4% |
| **3** | 0,0% | 100,0% | 20,0% | 80,0% | 5,0% | 95,0% | 1.3% | 98,7% |
| **4** | 0,0% | 100,0% | 30,0% | 70,0% | 7,5% | 92,5% | 1.9% | 98,1% |
| **5** | 0,0% | 100,0% | 50,0% | 50,0% | 10,0% | 90,0% | 2.5% | 97,5% |
| **6** | 0,0% | 100,0% | 70,0% | 30,0% | 12,5% | 87,5% | 3.8% | 96,2% |
| **7** | 0,0% | 100,0% | 90,0% | 10,0% | 15,0% | 85,0% | 4.4% | 95,6% |
| **8** | 0,0% | 100,0% | 100,0% | 0,0% | 20,0% | 80,0% | 5.0% | 95,0% |


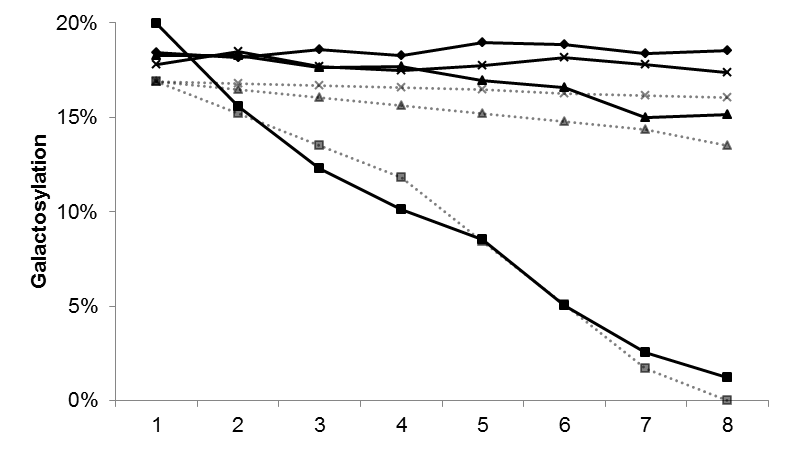


Figure S4 Results proof of concept study. Galactosylation levels of experiment 1 (diamond), experiment 2 (square), experiment 3 (triangle) and experiment 4 (cross). Dashed line in the same color indicate the theoretical values.


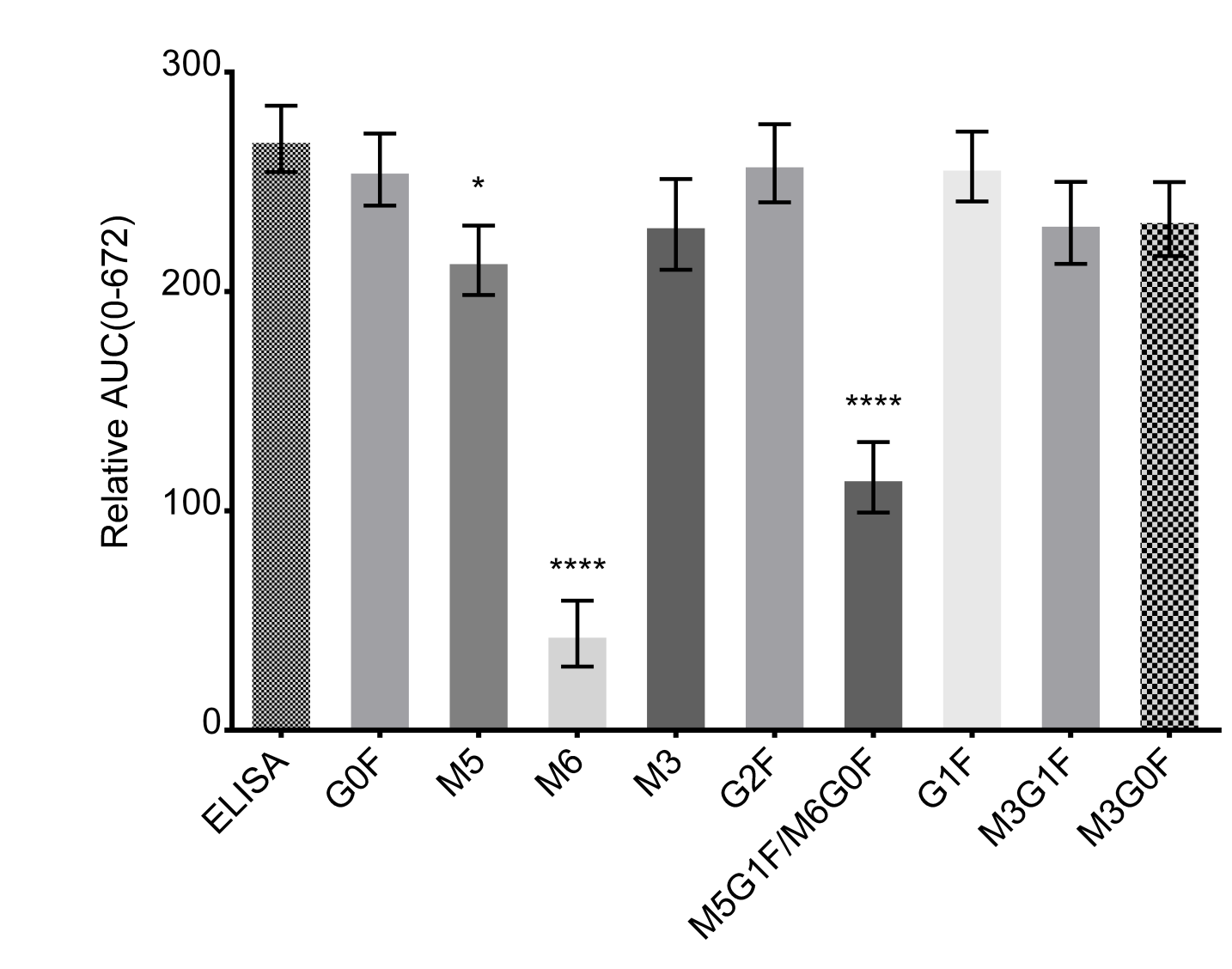


Figure S5 Relative AUCs (0-672) of the N-glycan and ELISA PK profiles with standard error. From unpaired t-tests statistically different N-glycan profiles (p<0.05) in comparison to the ELISA are marked. The number of stars indicates the level of significance.
